# Prevalence of adiposity and its association with sleep duration, quality, and timing among 9–12-year-old children in Guangzhou, China

**DOI:** 10.1016/j.je.2016.11.003

**Published:** 2017-06-13

**Authors:** Jiao Wang, Peymane Adab, Weijia Liu, Yajun Chen, Bai Li, Rong Lin, Wei Liu, Kar Keung Cheng, Miranda Pallan

**Affiliations:** a Department of Maternal and Child Health, School of Public Health, Sun Yat-Sen University, China; b Public Health, Epidemiology and Biostatistics, School of Health and Population Sciences, College of Medical and Dental Sciences, University of Birmingham, United Kingdom; c Department of School Health, Gangzhou Center for Disease Control and Prevention, China

**Keywords:** Sleep duration, Sleep quality, Bedtime, Adiposity, Children

## Abstract

**Background:**

Short sleep duration has been reported to be associated with obesity in children, but findings are not consistent. Since few studies have examined the relationship between more complex sleep characteristics and obesity, we examined the association between adiposity and self-reported sleep duration, bedtime, and sleep quality in 9–12-year-old Chinese children using multilevel mixed models.

**Methods:**

5518 children aged 9–12 years were recruited from 29 randomly selected primary schools in Guangzhou, China in 2014. Standardized questionnaires were used to obtain data to estimate sleep duration on typical weekdays and weekends. Sleep quality data were collected using the Children's Sleep Habits Questionnaire (CSHQ). Trained researchers undertook measurements of weight, height, and waist circumference (WC) for all participating children. Body mass index (BMI) z-scores were derived using the World Health Organization (WHO) child growth reference, and children were classified as overweight or obese using +1 and +2 SD as cut-offs, respectively. Percentage body fat (BF%) was calculated using bioelectrical impedance.

**Results:**

Longer sleep duration was inversely associated with BMI z-score (*β* = −0.16, *p* < 0.05), WC (*β* = −1.11, *p* < 0.05) and later bedtime was associated with higher BMI z-score (*β* = 0.03, *p* < 0.05), WC (*β* = 1.72, *p* < 0.001), and BF% (*β* = 0.15, *p* < 0.05) in multivariable multilevel mixed models, after adjustment for age, gender, physical activity, parental education level, and average monthly income. No association was seen between sleep quality and adiposity.

**Conclusion:**

Shorter sleep duration and later bedtime are associated with higher adiposity indices in early adolescents from southern China.

## Highlights

• Longer sleep duration was inversely associated with obesity in Chinese children.
• Later bedtime was positively associated with obesity in this population.
• No association between sleep quality and obesity was observed.

## Introduction

The prevalence of obesity has been increasing worldwide. Particularly, the prevalence of obesity and overweight are alarming in China, where economic transitions have resulted in changes to the traditional diet, increased sedentary lifestyles, and reduced physical activity.^1^, ^2^ Increased body weight may lead to many problems, such as psychosocial problems, lack of confidence, as well as chronic diseases, such as hypertension and type 2 diabetes. Complex factors contribute to childhood adiposity, including biological factors^3^ and lifestyle factors, such as sedentary lifestyle and intake of junk foods.^4^ However, in recent years, observational research in different age groups from the United States,^5^ Canada,^6^ and Australia,^7^ have reported that shorter sleep duration may be an additional risk factor associated with higher body mass index (BMI) among children.

Apart from this, there is increasing evidence showing that high-quality, adequate sleep is important for an overall healthy body,^5^, ^6^, ^7^ while sleep duration has been decreasing over time among children and adolescents. For example, national surveys in the United States have shown a decline in self-reported sleep duration among newborns to 10-year-olds over the past 50 years by 1.5–2 h,^8^ which may be attributable to changes in lifestyle, such as waking up early for school and late night activities.^9^ Sleep disturbance, characterized by disruptions in quantity, quality, or timing of sleep, frequently occur in children. Depending on the definitions of problematic sleep and methodologies employed, the reported prevalence of sleep disturbances in this age group varies from 20% to 45% in Western populations^10^, ^11^ and is towards the higher end^12^ or may be even higher in young children in China.^13^ However, few studies have been conducted on the relationship between more complex sleep characteristics and different obesity indices in China. One study has been undertaken in Shanghai^14^ on early adolescents, which found that short sleep duration was associated with higher adiposity indices.

In this study, we investigated the association between adiposity and self-reported sleep duration, sleep quality, and bedtime using different measures of adiposity in Chinese children aged 9–12 years.

## Methods

### Study design and subjects

The analysis presented in this paper comes from a sub-group of participants drawn from a larger cross-sectional study. The aim of the larger study was to determine the prevalence and risk factors for childhood overweight/obesity in primary school-aged children in Guangzhou. A multi-stage stratified cluster random sampling method was used to obtain a representative sample. Using a random number generator, five of the ten urban districts were first selected. Within each of the selected districts, schools were stratified by public (residents) or private (migrant) status, and six primary schools were randomly chosen, with a 2:1 ratio from each stratum. Within each school, two classes per year group (from grade 1 to 5) were randomly selected, from which all pupils (mainly age 6–12 years) were invited to take part. Children were excluded if they had serious health problems, including any physical and psychological condition that was felt by teaching staff to compromise their participation in the study (e.g., children with major disability or serious cognitive or psychological dysfunction). Permission for the study was not obtained for one of the private schools in our sampling frame, leaving 29 participating schools.

**Fig. 1:**
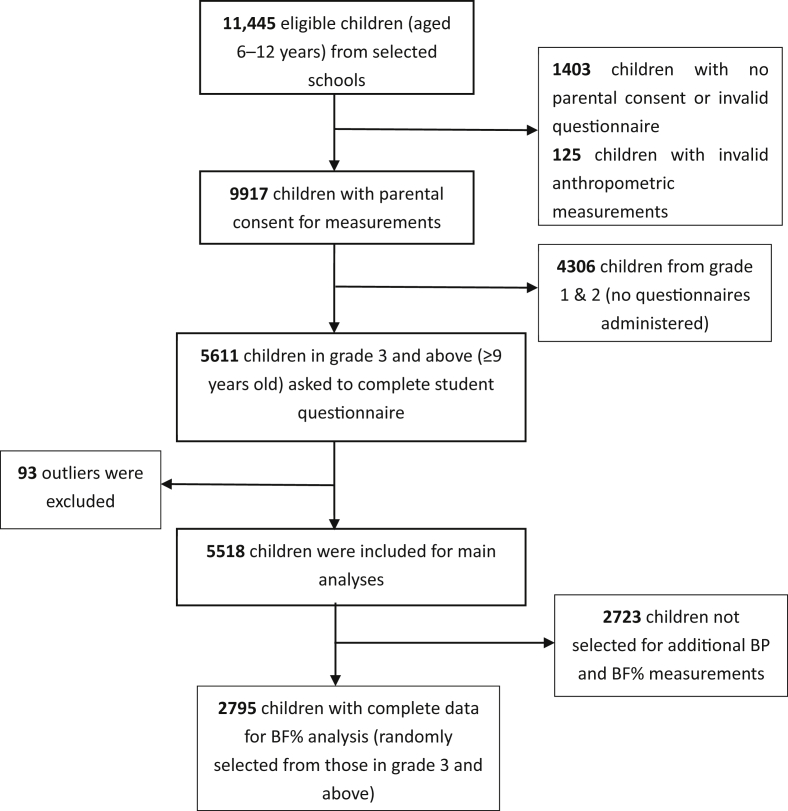
Flow chart of participants included in study.

Written informed consent was sought from the parents of 11,445 eligible children aged 6–12 years (on behalf of their children), resulting in 9917 participants (86.6% of eligible participants) (Fig. 1). Data collection took place from April to June 2014, with anthropometric measurements undertaken in school by trained research staff using standardized procedures and instruments. All children had measures of height and weight. The parents of all participating children were asked to complete a questionnaire that inquired about sociodemographic and lifestyle characteristics, and children in grade 3 and above (around 9 years old and above) were asked to complete a student questionnaire (*n* = 5518). More detailed measures, such as blood pressure and percentage body fat (BF%), were undertaken on a randomly selected sub-sample across all school grades (approximately 50% of the total sample). The number of children with complete data for this analysis was 2795 (1500 boys and 1295 girls aged 9–12 years).

### Data collection and measurements

#### Socio-demographic questionnaire

Data on parental demographic characteristics, such as parental educational level (junior high school and below/middle high school/university and above) and average household income, were collected through the parent/guardian questionnaire.

Physical activity was assessed using the following item: *“Please estimate on average, how long your child spends on each of the following activities each day.”* Physical activity level was classified into four categories: sitting (e.g., classroom work, homework, reading, watching TV, playing computer, sewing, eating, or sitting in car or bus); light physical activities (e.g., getting dressed or undressed, tidying a room, feeding or playing with pets, imaginary play, playing a musical instrument, or cooking); moderate physical activity (e.g., playing in the garden, playground games, walking, bicycling [slow/moderate speeds], swimming for fun, dancing, or gymnastics); and vigorous physical activity (e.g., running, bicycling [fast speeds], football, tennis, rugby, roller-skating, or length swimming).

The student questionnaires included questions on the following: district, school, grade, class, gender, age, and sleep information. The completed questionnaires were reviewed by trained staff and uploaded into the database.

Children were asked to report evening bedtime and wake-up time for weekdays and weekends in the preceding week. The average sleep duration was calculated as the weighted average of weekday and weekend sleep durations using the formula: [(weekday sleep duration ×5)+(weekend sleep duration ×2)]/7. The calculated average sleep duration was then classified into four groups based on the mean and standard deviation of the total sample (*n* = 5518). Groups were defined as: shortest (≤−1 standard deviation [SD]; *n* = 734, range: 6.25–8.61 h); shorter (−1 SD to the mean; *n* = 2181, range: 8.61–9.61 h); longer (mean to 1 SD; *n* = 1912, range: 9.61–10.61 h); and longest (≥1 SD; *n* = 691, range: 10.61–13.09 h) sleep duration (eFig. 1). Those who reported sleep duration >±3 SD from the median were excluded as implausible values. Twenty-one items from the Children's Sleep Habit Questionnaire (CHSQ)^15^ were included in the parents' questionnaire, covering five subscales (bedtime resistance, sleep onset delay, sleep duration, night waking, and daytime sleepiness). A total sleep disturbance score was calculated from the items, from 20 to 60, with a higher score indicative of greater sleep disturbance. The Chinese version of the CSHQ was developed via translation and back translation and has been used previously with proven excellent sensitivity and reliability.^16^

#### Anthropometric measurements

Height and weight were measured with subjects wearing light clothing and without shoes. Height was recorded to the nearest 0.1 cm with a TGZ type height tester (Dalian, China). Weight was measured to the nearest 0.1 kg using an electronic scale (JH-1993T; Weighing Apparatus Co. Ltd., Dalian, China). Body mass index (BMI; $\left[weight\left(kg\right)\right]/{\left[height\left(m\right)\right]}^{2}$) was calculated, and BMI standard deviation scores (BMI z-score) were derived using the age (calculated by subtracting the date of birth from the date of examination) and sex-specific World Health Organization (WHO) growth reference for school-aged children, which were further classified as non-overweight (≤1 SD), overweight (>1 SD) and obese (>2 SD).^17^ Waist circumference (WC) was measured to the nearest 0.1 cm at the midpoint between the bottom of the rib cage and the top of the iliac crest at the end of exhalation. A MyoTape waistline measurer was employed with the subject standing without clothing covering the waist area. WC measurements were performed twice, and the average of the two measurements was calculated for use in the analyses. Waist-to-height ratio (WHtR) was calculated by dividing average WC (cm) by height (cm).

In a subsample of 2795 subjects, bioelectrical impedance analysis was performed to assess body composition using a single-frequency ImpediMed machine (ImpDF50; Impedimed Pty Ltd, Queensland, Australia). Leads were attached to each child's wrists and ankles when he or she was lying down (after voiding the bladder). From the impedance recording, fat free mass (FFM) was calculated using a specific equation derived from Chinese Center for Disease Control and Prevention^18^ ($FFM=0.290\;weight+2.222\;sex+0.427\;height^{2}/impedance+1.547$), which is validated in Chinese children, and from this percentage body fat (BF%) was calculated [(weight−FFM)/weight×100%].

### Statistical analysis

Descriptive statistics were used to examine the relationship between sleep and anthropometric variables by age and sex. Generalized linear mixed models (GLMM) were developed to assess the relationship between measures of adiposity as outcomes (BMI z-score, WC, WHtR, and BF%), and sleep variables (sleep duration, sleep disturbance score, and bedtime), adjusted for potential confounders. School was included as a random effect in all models, as the likelihood ratio (LR) test statistic was significant at the 5% level. Separate models were developed for each sleep and adiposity variable, and these were developed as follows: model 1 was unadjusted; model 2 was fitted with individual level variables (age and gender); model 3 adjusted for age, gender, physical activities, parental education level, and average income monthly. Average sleep duration was also included in model 3 when the explanatory variables were the sleep disturbance score and bedtime in order to test if the sleep disturbance score or bedtime were independent factors for childhood adiposity. Finally, we reported the prevalence of overweight and obesity using other criteria: a) using the Chinese national reference norm introduced by the working group on obesity in China (WGOC),^19^ and b) the criteria introduced by international obesity taskforce (IOTF), instead of the WHO 2007 child growth standard,^20^ to be comparable with other publications (eTable 1). All statistical analyses were performed using SPSS (version 21.0, SPSS, Inc., Chicago, IL, USA), and *P* values of less than 0.05 were considered to be statistically significant.

### Standard protocol approvals, registrations, and patient consent

Written informed consent was obtained from all participants and guardians of participants in the study. The study was approved by the Ethics Committee of Guangzhou Center for Disease Control and Prevention and the University of Birmingham Ethics Committee. Permissions to conduct the study and contact the sampled schools were granted by the Departments of Education and Health.

## Results

The descriptive characteristics of the participants are shown in Table 1. The mean age was 10.24 years (SD, 0.96), and 53.9% were boys. Compared with girls, boys had significantly higher weight, BMI z-score, WC, and WHtR, but lower BF%. The prevalence of overweight and obesity was 13.2% and 7.3%, respectively, using WHO criteria; 9.5% and 5.5%, respectively, using WGOC criteria; and 12.0% and 3.3%, respectively, using IOTF criteria (eTable 1). The mean reported sleep duration was 9.61 h (SD, 1.00), being shorter during weekdays (9.25 h) compared with weekend days (9.97 h). The average reported bedtime was 21:58 (SD, 59 min), being slightly earlier in girls compared with boys, and the mean total sleep disturbance score was 29.24 (SD, 4.92). Approximately children spend 7.21 h, 4.75 h, 1.74 h, and 1.52 h on sitting, light physical activity, moderate physical activity, and vigorous physical activity, respectively, reported among children each day.

**Table 1:** Participant characteristics stratified by gender.

	Total Mean (SD) or n (%)	Boys Mean (SD) or n (%)	Girls Mean (SD) or n (%)	*P* value
**Participant & Family Characteristics**				
N (%)^b^	5518	2975 (53.9)	2543 (46.1)	
Age, years	10.24 (0.96)	10.30 (0.96)	10.18 (0.96)	**<0.001**
Height, cm	138.85 (8.26)	138.62 (7.98)	139.12 (8.57)	**<0.001**
Weight, kg	33.05 (8.50)	33.80 (8.90)	32.18 (7.93)	**<0.001**
BMI, kg/m^2^	16.94 (2.99)	17.37 (3.18)	16.43 (2.66)	**<0.001**
BMI z-score	−0.14 (1.32)	0.07 (1.40)	−0.39 (1.18)	**<0.001**
Overweight, %^b^	745 (13.2)	483 (15.7)	262 (10.1)	**<0.001**
Obesity, %^b^	411 (7.3)	338 (11.0)	73 (2.8)	**<0.001**
WC, cm	58.35 (7.75)	60.04 (8.41)	56.40 (6.38)	**<0.001**
WHtR	0.42 (0.05)	0.43 (0.05)	0.41 (0.04)	**<0.001**
BF%^a^	24.30 (7.44)	20.44 (7.52)	28.77 (4.06)	**<0.001**
Physical activity				**<0.001**
Sitting, hours per day	7.21 (2.75)	7.31 (2.80)	7.10 (2.68)	0.78
Light physical activities, hours per day	4.75 (1.65)	4.72 (1.64)	4.78 (1.67)	0.73
Moderate physical activity, hours per day	1.74 (1.24)	1.74 (1.24)	1.74 (1.24)	0.26
Vigorous physical activity, hours per day	1.52 (1.22)	1.59 (1.21)	1.45 (1.22)	0.49
Maternal educational level				**0.04**
Junior high school and below	2328 (43.6)	1287 (44.9)	1041 (42.1)	
Middle high school	2288 (42.8)	1232 (43.0)	1056 (42.7)	
University and above	724 (13.6)	349 (12.2)	375 (15.2)	
Paternal educational level				**0.02**
Junior high school and below	2746 (51.3)	1526 (53.3)	1220 (49.0)	
Middle high school	2005 (37.5)	1046 (36.6)	959 (38.5)	
University and above	598 (11.2)	289 (10.1)	309 (12.4)	
Average income monthly, Yuan per person	2669.3 (5117.8)	2636.9 (5203.3)	2706.9 (5157.6)	0.07
**Sleep variables**				
Average nocturnal sleep duration, hours per night	9.61 (1.00)	9.58 (1.08)	9.64 (0.90)	0.88
Weekday sleep duration, hours per night	9.25 (1.00)	9.25 (1.04)	9.24 (0.94)	0.17
Weekend sleep duration, hours per night	9.97 (1.50)	9.92 (1.65)	10.04 (1.28)	0.29
Average bedtime	21:58 (59 min)	21:59 (58 min)	21:56 (50 min)	0.20
Weekday sleep bedtime	21:32 (57 min)	21:31 (55 min)	21:31 (51 min)	0.97
Weekend sleep bedtime	22:23 (86 min)	22:26 (83 min)	22:20 (66 min)	0.07
Total sleep disturbance score	29.24 (4.92)	29.15 (4.84)	29.31 (5.00)	0.20

BF%, body fat percentage; BMI, body mass index; SD, standard deviation; WC, waist circumference; WHtR, waist-to-height ratio.

*P* values were calculated by chi-squared tests for categorical variables and two-sample *t-*tests for continuous variables between genders. Bold type indicates *p* < 0.05.

Data marked with “^b^” as frequencies, all others were presented as means (standard deviations).

a Based on a sub-sample of 2795 participants.

Results of the models examining the association between self-reported sleep and adiposity measures are shown in Table 2. Children with longer sleep duration had significantly lower BMI z-scores, WC, and WHtR than those with the shortest sleep duration. The association was attenuated in the adjusted models but remained statistically significant (*β* = −0.16 for BMI z-score and *β* = −1.11 for WC as the outcome). Later bedtime was associated with increased BMI-z score (*β* = 0.03), WC (*β* = 1.72), and BF% (*β* = 0.15) after adjustment for age, gender, physical activities, parental education level, average income monthly, and average sleep duration. There was also no relationship between the sleep disturbance score and any measure of adiposity.

**Table 2:** Associations between self-reported sleep and adiposity measures for the unadjusted and adjusted liner multilevel regression models.

BMI z-score (*n* = 5518)^a^		Unadjusted model (model 1)*		Individual factor-adjusted model (model 2)**		Fully-adjusted model (model 3)***	
		Coefficient (95% CI)	*P* value	Coefficient (95% CI)	*P* value	Coefficient (95% CI)	*P* value
Sleep duration, hours	shortest	0.00 (reference)		0.00 (reference)		0.00 (reference)	
	shorter	**−0.13 (−0.20, −0.06)**	**<0.001**	−0.09 (−0.20, 0.024)	0.13	−0.10 (−0.24, 0.04)	0.12
	longer	**−0.25 (−0.33, −0.18)**	**<0.001**	**−0.18 (−0.30, −0.07)**	**0.001**	**−0.16 (−0.30, −0.01)**	**0.03**
	longest	−0.11 (−0.23, 0.01)	0.07	−0.07 (−0.22, 0.07)	0.31	−0.08 (−0.26, 0.10)	0.46
Sleep disturbance score		**−0.005 (−0.007, −0.003)**	**<0.001**	−0.001 (−0.007, 0.006)	0.82	0.000 (−0.008, 0.009)	0.91
Bedtime, hours		**−0.006 (−0.009, −0.003)**	**0.004**	**0.03 (0.01, 0.05)**	**0.001**	**0.03 (0.005, 0.05)**	**0.02**
WC (*n* = 5518)^a^							
Sleep duration, hours	shortest	0.00 (reference)		0.00 (reference)		0.00 (reference)	
	shorter	**−1.26 (−1.91, −0.61)**	**<0.001**	**−0.87 (−1.50, −0.25)**	**0.006**	−0.78 (−1.57, −0.001)	0.05
	longer	**−2.12 (−2.80, −1.45)**	**<0.001**	**−1.36 (−2.01, −0.72)**	**<0.001**	**−1.11 (−1.92, −0.31)**	**0.007**
	longest	**−1.74 (−2.60, −0.88)**	**<0.001**	**−0.91 (−1.73, −0.09)**	**0.03**	−0.66 (−1.69,0.37)	0.21
Sleep disturbance score		**−0.07 (−0.11, −0.02)**	**0.004**	−0.03 (−0.08,0.01)	0.18	−0.003 (−0.06,0.05)	0.90
Bedtime, hours		**0.86 (0.64, 1.08)**	**<0.001**	**0.53 (0.32, 0.73)**	**<0.001**	**1.72 (1.59, 1.84)**	**<0.001**
WHtR (*n* = 5518)^a^							
Sleep duration, hours	shortest	0.00 (reference)		0.00 (reference)		0.00 (reference)	
	shorter	**−0.009 (−0.013, −0.005)**	**<0.001**	**−0.006 (−0.010, −0.002)**	**0.004**	**−**0.003 (**−**0.007, 0.007)	0.30
	longer	**−0.012 (−0.017, −0.008)**	**<0.001**	**−0.009 (−0.013, −0.005)**	**<0.001**	**−**0.004 (**−**0.010, −0.002)	0.07
	longest	−0.006 (−0.011, 0.000)	0.04	**−**0.005 (**−**0.010, 0.000)	0.05	**−**0.000 (**−**0.007, 0.007)	0.96
Sleep disturbance score		0.000 (−0.000, 0.000)	0.66	0.000 (−0.000, 0.000)	0.84	0.000 (−0.000, 0.001)	0.58
Bedtime, hours		0.001 (0.000, 0.002)	0.07	**0.002 (0.001, 0.003)**	**0.003**	0.003 (0.001, 0.004)	0.002
BF% (*n* = 2795)^a^							
Sleep duration, hours	shortest	0.00 (reference)		0.00 (reference)		0.00 (reference)	
	shorter	**1.61 (0.77, 2.44)**	**<0.001**	0.17 (−0.51, 0.85)	0.62	0.28 (−0.59, 1.14)	0.53
	longer	**1.24 (0.37, 2.12)**	**<0.001**	−0.25 (−0.96, 0.45)	0.48	0.16 (−0.74, 1.06)	0.73
	longest	0.17 (−0.95, 1.30)	0.76	−0.27 (−1.17, 0.63)	0.56	−0.06 (−1.21,1.09)	0.92
Sleep disturbance score		0.01 (−0.05, 0.07)	0.78	0.02 (−0.03, 0.07)	0.36	0.04 (−0.02, 0.09)	0.21
Bedtime, hours		**1.10 (1.07,1.13)**	**<0.001**	**0.14 (0.033, 0.25)**	**0.01**	**0.15 (0.01, 0.29)**	**0.04**

BF%, body fat percentage; BMI, body mass index; CI, confidence interval; WC, waist circumference; WHtR, waist-to-height ratio.

Significant *P* values (α = 0.05) are bolded.

*Model 1: unadjusted.

**Model 2: adjusted for age and gender.

***Model 3: adjusted for age, gender, and physical activities, parental education level, and average monthly income (as well as sleep duration in the models with sleep disturbance and bedtime as the main predictor variable).

a Number of participants included in the fully adjusted model.

## Discussion

In this study, with a representative sample of 9–12-year-old children from an urban Chinese city, we found that longer sleep duration was inversely associated and later bedtime was positively associated with all measures of adiposity, while no association between sleep quality and adiposity was observed.

The mean sleep duration in this study (9.61 h) was very similar to that previously reported in similar aged Chinese children (9.46 h),^14^ but the duration in our study was at the low end of that recommended for this age group^21^ and lower than that reported for children of similar ages in Western countries. An American study reported a mean sleep duration of 10.15 h in elementary school children (kindergarten through to grade 4),^22^ and a Swiss study reported a mean sleep duration of 10.2 h in 10-year-old children.^23^ Usual bedtime was also relatively later than expected in this population, which, without a compensatory delay in waking time, may explain the shorter sleep duration.^24^ These differences are likely to be cultural and due to the overemphasis on studying and academic achievement in China.^25^ Other observations of decreasing sleep duration with increasing age and longer weekend than weekday sleep duration in our study are similar to findings in other studies.^26^

Our finding of an association between shorter sleep duration and obesity is consistent with previous studies in many settings, including prospective cohort studies,^27^, ^28^ which have shown a consistent negative linear association between baseline habitual sleep duration and later obesity. We found that this association was consistent for nearly all measures of adiposity and have confirmed this association in a population with shorter overall sleep duration in China. Our findings agree with those from a recent study in Shanghai,^14^ which also found an association between shorter sleep duration and risk of different measures of obesity in 10-year old children. Our findings add to those of the previous study by examining the association in a broader age range of pre-adolescents (9–12 years old) and by additionally examining the association between bedtime and sleep quality with adiposity. Although the differences in BMI z-score seem small, there is evidence that even a change in BMI z-score of 0.1 units^29^, ^30^ is clinically important and associated with significant change in health outcomes. Experimental studies, especially in mammals,^31^ have demonstrated potential biological mechanisms to explain this relationship, through metabolic derangement leading to decreased leptin, elevated ghrelin, and resultant increased appetite.^32^ Behavioral mechanisms have also been proposed, with tiredness resulting from short sleep duration leading to restricted physical activity^33^; and more waking hours providing additional opportunities to eat.^34^

Independently of sleep duration, there are conflicting reports on the association between poor sleep quality and obesity in children. Some studies have found such an association,^35^, ^36^, ^37^ hypothesizing hormonal mechanisms (i.e., increased cortisol and decreased growth hormone secretion) due to alterations in the sleep architecture. In contrast to our findings, a cross-sectional Canadian study of 10-year old children^33^ found that objectively-measured sleep duration and bedtime were not associated, whereas sleep efficiency (a measure of quality) was inversely associated with measures of adiposity. This may partly be due to the differences in the way sleep duration, quality, and bedtime were assessed, but is also likely due to the different populations and differences in sleep and physical activity habits. On the other hand, other similar studies^38^, ^39^ found no association between sleep quality and adiposity. A longitudinal study of primary-school-aged children in Belgium,^35^ like our study, reported an inverse relationship between sleep duration and central adiposity but no relationship between sleep quality and measures of adiposity. These contrasting findings, from distinct cultural and racial settings, suggest that the association between sleep parameters and adiposity are more complex and need further investigation, preferably with objective measures of sleep, comprehensive markers of adiposity, and longitudinal assessment.

More recently, a few studies have reported associations between obesity and late bedtime,^28^, ^40^, ^41^, ^42^, ^43^ with some^41^, ^42^, ^43^ reporting this to be independent of sleep duration. Ours is the first study to report an association between later bedtime and a variety of measures of adiposity, independent of sleep duration and after adjusting for potential confounding factors in Guangzhou children. The potential mechanism for this finding is likely to be at least partly behavioral. Obesogenic behaviors, such as TV watching and late-night snacking, are more common in the later evening. However, it is also possible that circadian phase-delay may play a role in explaining the risk for overweight/obesity, particularly given research suggesting the importance of circadian clocks in metabolism and obesity.^44^ Taken together, study findings suggest the need to further explore the multiple pathways through which bedtime influences obesity.

Our study extends the findings from a study in Shanghai^14^ indicating that there are associations between sleep characteristics (duration and bedtime) and multiple adiposity indices in a large sample of Chinese children. However, we found no evidence that sleep quality, independent of sleep duration, was associated with adiposity. Whilst most studies have used BMI, a few recent studies examined other indices, such as skinfold thickness^45^, ^46^ and waist circumference.^47^ This is important, as it suggests that shorter sleep duration is associated with both the amount and distribution of body fat, specifically with central obesity.

Our study also has several potential limitations. First, sleep duration reported by children may be inaccurate. However, there is research to suggest that reported sleeping hours may be uniformly overestimated,^48^ which means that this would not have a great effect on the observed relationship between short sleep duration and obesity. Second, the relationship may be confounded by diet, which was not assessed in this study. A further limitation is the cross-sectional study design, which meant that we were unable to determine a temporal relationship between sleep duration and adiposity.

In summary, the findings of this study support the previously observed relationship between sleep duration and adiposity. Children with longer sleep duration, on average, have BMI z-scores 0.16 units lower and WCs 1.11 cm lower than those with shortest sleep duration, and for every hour later that a child goes to bed, their BMI z-score is 0.03 units greater and WC is 1.72 cm greater on average. Although these differences in BMI z-score seem small, there is evidence that even a change in BMI z-score of 0.1 units is clinically important and associated with significant change in health outcomes. This study contributes to the existing evidence for sleep duration as a risk factor for obesity in childhood, and later bedtime as an additional risk factor, independent of sleep duration. Further studies are required to determine the causal mechanisms for these relationships.

## Conflicts of interest

None declared.

## References

[1] Lobstein T., Baur L., Uauy R. (2004). Obesity in children and young people: a crisis in public health. Obes Rev.

[2] Wu Y. (2006). Overweight and obesity in China the once lean giant has a weight problem that is increasing rapidly. BMJ.

[3] Silventoinen K., Rokholm B., Kaprio J., Sorensen T.I. (2010). The genetic and environmental influences on childhood obesity: a systematic review of twin and adoption studies. Int J Obes (Lond).

[4] Andersen R.E., Crespo C.J., Bartlett S.J., Cheskin L.J., Pratt M. (1998). Relationship of physical activity and television watching with body weight and level of fatness among children: results from the Third National Health and Nutrition Examination Survey. JAMA.

[5] Boeke C.E., Storfer-Isser A., Redline S., Taveras E.M. (2014). Childhood sleep duration and quality in relation to leptin concentration in two cohort studies. Sleep.

[6] Chaput J.P., Brunet M., Tremblay A. (2006). Relationship between short sleeping hours and childhood overweight/obesity: results from the 'Quebec en Forme' Project. Int J Obes (Lond).

[7] Eisenmann J., Ekkekakis P., Holmes M. (2006). Sleep duration and overweight among Australian children and adolescents. Acta Paediatr.

[8] Mindell J.A., Meltzer L.J., Carskadon M.A., Chervin R.D. (2009). Developmental aspects of sleep hygiene: findings from the 2004 national sleep foundation sleep in America poll. Sleep Med.

[9] Magee C., Caputi P., Iverson D. (2014). Lack of sleep could increase obesity in children and too much television could be partly to blame. Acta Paediatr.

[10] Fricke-Oerkermann L., Pluck J., Schredl M. (2007). Prevalence and course of sleep problems in childhood. Sleep.

[11] Hiscock H., Canterford L., Ukoumunne O.C., Wake M. (2007). Adverse associations of sleep problems in Australian preschoolers: national population study. Pediatrics.

[12] Guo L., Deng J., He Y. (2014). Prevalence and correlates of sleep disturbance and depressive symptoms among Chinese adolescents: a cross-sectional survey study. BMJ Open.

[13] Liu Z., Wang G., Geng L., Luo J., Li N., Owens J. (2014). Sleep patterns, sleep disturbances, and associated factors among Chinese urban kindergarten children. Behav Sleep Med.

[14] Jiang Y.R., Spruyt K., Chen W.J. (2015). Associations between parent-reported sleep duration and adiposity in Chinese early adolescents. J Public Health-UK.

[15] Owens J.A., Spirito A., McGuinn M. (2000). The Children's Sleep Habits Questionnaire (CSHQ): psychometric properties of a survey instrument for school-aged children. Sleep.

[16] Li S.H., Jin X.M., Shen X.M. (2007). Development and psychometric properties of the Chinese version of Children's sleep habits questionnaire. Zhonghua Er Ke Za Zhi.

[17] de Onis M.Oabe (2007). Development of a WHO growth reference for school-aged children and adolescents. B World Health Organ.

[18] Wang J., Wang X., Hu X., Ma G. (2008). Study on the formulas predicted for body fat of children and adolescents by using bioelectrical impedance analysis. Wei Sheng Yan Jiu.

[19] Ji C.Y. (2005). Report on childhood obesity in China (1)–body mass index reference for screening overweight and obesity in Chinese school-age children. Biomed Environ Sci.

[20] Cole T.J., Bellizzi M.C., Flegal K.M., Dietz W.H. (2000). Establishing a standard definition for child overweight and obesity worldwide: international survey. BMJ.

[21] Hirshkowitz M., Whiton K., Albert S.M. (2015). National Sleep Foundation's sleep time duration recommendations: methodology and results summary. Sleep Health.

[22] Liu X., Liu L., Owens J.A., Kaplan D.L. (2005). Sleep patterns and sleep problems among schoolchildren in the United States and China. Pediatrics.

[23] Iglowstein I., Jenni O.G., Molinari L., Largo R.H. (2003). Sleep duration from infancy to adolescence: reference values and generational trends. Pediatrics.

[24] Kohyama J., Shiiki T., Hasegawa T. (2000). Sleep duration of young children is affected by nocturnal sleep onset time. Pediatr Int.

[25] Jiang F., Zhu S., Yan C., Jin X., Bandla H., Shen X. (2009). Sleep and obesity in preschool children. J Pediatr.

[26] Thorleifsdottir B., Bjornsson J.K., Benediktsdottir B., Gislason T., Kristbjarnarson H. (2002). Sleep and sleep habits from childhood to young adulthood over a 10-year period. J Psychosom Res.

[27] Agras W.S., Hammer L.D., McNicholas F., Kraemer H.C. (2004). Risk factors for childhood overweight: a prospective study from birth to 9.5 years. J Pediatr.

[28] Snell E.K., Adam E.K., Duncan G.J. (2007). Sleep and the body mass index and overweight status of children and adolescents. Child Dev.

[29] Kirk S., Zeller M., Claytor R., Santangelo M., Khoury P.R., Daniels S.R. (2005). The relationship of health outcomes to improvement in BMI in children and adolescents. Obes Res.

[30] Kolsgaard M.L., Joner G., Brunborg C., Anderssen S.A., Tonstad S., Andersen L.F. (2011). Reduction in BMI z-score and improvement in cardiometabolic risk factors in obese children and adolescents. The Oslo Adiposity Intervention Study - a hospital/public health nurse combined treatment. BMC Pediatr.

[31] Garaulet M., Gomez-Abellan P. (2014). Timing of food intake and obesity: a novel association. Physiol Behav.

[32] Taheri S., Lin L., Austin D., Young T., Mignot E. (2004). Short sleep duration is associated with reduced leptin, elevated ghrelin, and increased body mass index. PLoS Med.

[33] Taheri S. (2006). The link between short sleep duration and obesity: we should recommend more sleep to prevent obesity. Arch Dis Child.

[34] Bel S., Michels N., De Vriendt T. (2013). Association between self-reported sleep duration and dietary quality in European adolescents. Br J Nutr.

[35] Bawazeer N.M., Al-Daghri N.M., Valsamakis G. (2009). Sleep duration and quality associated with obesity among Arab children. Obes (Silver Spring).

[36] Gupta N.K., Mueller W.H., Chan W., Meininger J.C. (2002). Is obesity associated with poor sleep quality in adolescents?. Am J Hum Biol.

[37] Mcneil J., Tremblay M.S., Leduc G. (2015). Objectively-measured sleep and its association with adiposity and physical activity in a sample of Canadian children. J Sleep Res.

[38] Lumeng J.C., Somashekar D., Appugliese D., Kaciroti N., Corwyn R.F., Bradley R.H. (2007). Shorter sleep duration is associated with increased risk for being overweight at ages 9 to 12 years. Pediatrics.

[39] Michels N., Verbeiren A., Ahrens W., De Henauw S., Sioen I. (2014). Children's sleep quality: relation with sleep duration and adiposity. Public Health.

[40] Sekine M., Yamagami T., Handa K. (2002). A dose-response relationship between short sleeping hours and childhood obesity: results of the Toyama Birth Cohort Study. Child care, health Dev.

[41] Jarrin D.C., McGrath J.J., Drake C.L. (2013). Beyond sleep duration: distinct sleep dimensions are associated with obesity in children and adolescents. Int J Obes (Lond).

[42] Golley R.K., Maher C.A., Matricciani L., Olds T.S. (2013). Sleep duration or bedtime? Exploring the association between sleep timing behaviour, diet and BMI in children and adolescents. Int J Obes (Lond).

[43] Olds T.S., Maher C.A., Matricciani L. (2011). Sleep duration or bedtime? Exploring the relationship between sleep habits and weight status and activity patterns. Sleep.

[44] Bray M.S., Young M.E. (2007). Circadian rhythms in the development of obesity: potential role for the circadian clock within the adipocyte. Obes Rev.

[45] Padez C., Mourao I., Moreira P., Rosado V. (2005). Prevalence and risk factors for overweight and obesity in Portuguese children. Acta Paediatr.

[46] Ozturk A., Mazicioglu M., Poyrazoglu S., Cicek B., Gunay O., Kurtoglu S. (2009). The relationship between sleep duration and obesity in Turkish children and adolescents. Acta Paediatr.

[47] Hitze B., Bosy-Westphal A., Bielfeldt F. (2009). Determinants and impact of sleep duration in children and adolescents: data of the Kiel Obesity Prevention Study. Eur J Clin Nutr.

[48] Chen W.J., Li F., Li S.H. (2012). Comparative study of children's sleep evaluation methods. Zhonghua Er Ke Za Zhi.

