# Supplementary material for: Prevalence of adiposity and its association with sleep duration, quality, and timing among 9–12-year-old children in Guangzhou, China
Source: J Epidemiol. 2017 Jun 13;27(11):531–7. doi: 10.1016/j.je.2016.11.003 (PMC5608611; doi:10.1016/j.je.2016.11.003)
Supplement: Supplementary file 1 [file mmc1.pdf]

eFigure 1. Frequency polygon of sleep duration in early adolescents (n=5518). The solid line indicates the median of sleep duration and the dotted lines indicate 1 standard deviation.

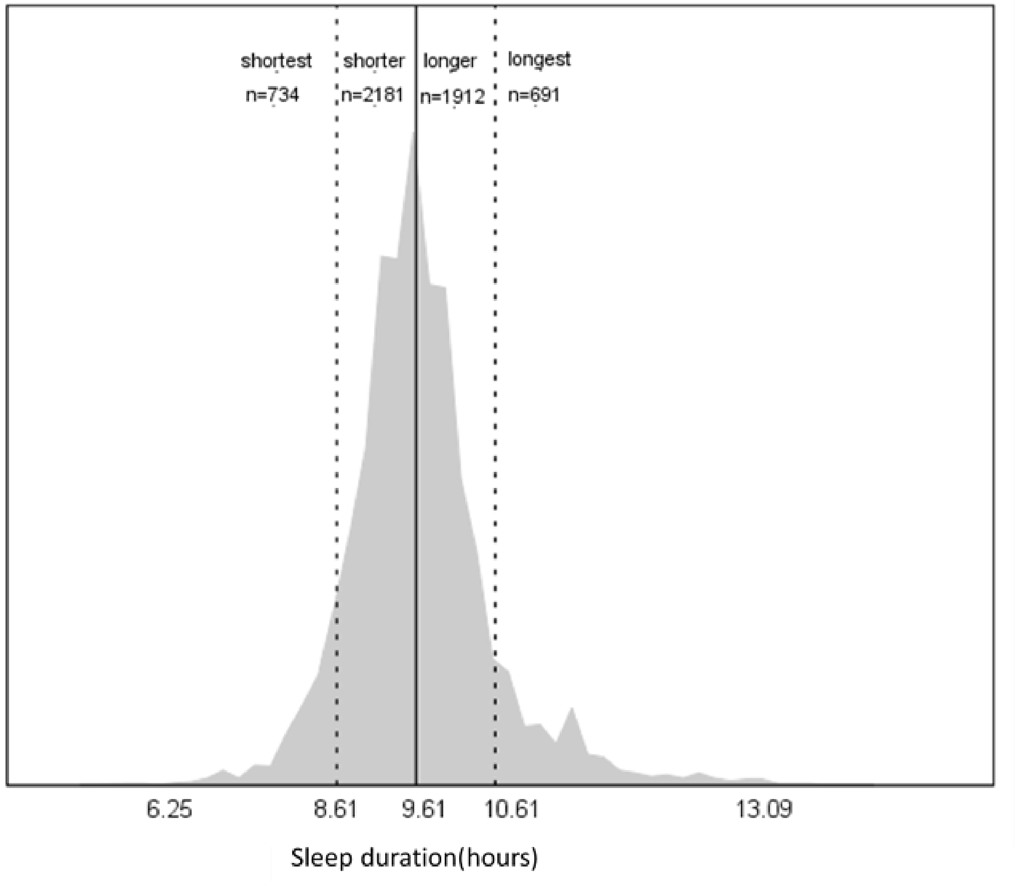

eTable 1. Prevalence of overweight and obesity stratified by gender using other criteria among 9–12 aged children in Guangzhou, China

	Total<br>n (%)	Boys<br>n (%)	Girls<br>n (%)	P value
Overweight[a]	534 (9.5)	392 (12.9)	142 (5.5)	**<0.001**
Obesity[a]	303 (5.5)	220 (7.2)	83 (3.2)	**<0.001**
Overweight[b]	675 (12.0)	444 (14.6)	231 (9.0)	**<0.001**
Obesity[b]	187 (3.3)	148 (4.9)	29 (1.5)	**<0.001**

[a] Calculated using Working Group on Obesity in China criterion.

[b] Calculated using International Obesity Task Force criterion.

eTable 2. Associations between bedtime (categorized) and adiposity measures for the unadjusted and adjusted liner multilevel regression models

		Unadjusted model (model 1) *		Individual factor-adjusted model (model 2) **		Fully adjusted model (model 3) ***	
BMI z-score (n=5518) [a]		Coefficient (95% CI)	P value	Coefficient (95% CI)	P value	Coefficient (95% CI)	P value
Bedtime	Before 21:45min	0.00 (reference)		0.00 (reference)		0.00 (reference)	
	21:45min–22:15min	−0.02 (−0.11, 0.06)	0.59	0.04 (−0.05, 0.13)	0.34	0.04 (−0.06, 0.15)	0.43
	After 22:15min	**0.09 (0.02,0.17)**	**0.02**	**0.13 (0.05, 0.21)**	**0.002**	**0.12 (0.02,0.22)**	**0.02**
WC (n=5518) [a]							
Bedtime	Before 21:45min	0.00 (reference)		0.00 (reference)		0.00 (reference)	
	21:45min–22:15min	**0.75 (0.24,1.27)**	**0.004**	0.32 (−0.17,0.80)	0.20	0.23 (−0.36,0.81)	0.45

	After 22:15min	**1.60 (1.11,2.08)**	**<0.001**	**0.79 (0.33,1.25)**	**<0.001**	**0.64 (0.08,1.20)**	**0.02**
WHtR (n=5518) [a]							
Bedtime	Before 21:45min	0.00 (reference)		0.00 (reference)		0.00 (reference)	
	21:45min–22:15min	0.002 (−0.001, 0.005)	0.21	0.001 (−0.002, 0.004)	0.37	0.001 (−0.003, 0.004)	0.75
	After 22:15min	**0.007 (0.004, 0.01)**	**<0.001**	**0.005 (0.002,0.007)**	**0.002**	0.003 (−0.000, 0.007)	0.06
BF% (n=2795) [a]							
Bedtime	Before 21:45min	0.00 (reference)		0.00 (reference)		0.00 (reference)	
	21:45min–22:15min	0.42 (−0.33,1.17)	0.27	0.23 (−0.37,0.84)	0.45	0.14 (−0.57,0.85)	0.69
	After 22:15min	−1.26 (−0.88,0.63)	0.75	**0.64 (0.03,1.26)**	**0.04**	0.55 (−0.18,1.27)	0.14

BF%, body fat percentage; BMI, body mass index; CI, confidence interval; WC, waist circumference; WHtR, waist-to-height ratio;

Significant P-values (α=0.05) are bolded.

*Model 1: unadjusted

**Model 2: adjusted for age and gender

***Model 3: adjusted for age, gender, and physical activities, parental education level, average income monthly, and sleep duration

[a] Number of participants included in the fully adjusted model
